# NS5ATP9 Contributes to Inhibition of Cell Proliferation by Hepatitis C Virus (HCV) Nonstructural Protein 5A (NS5A) via MEK/Extracellular Signal Regulated Kinase (ERK) Pathway

**DOI:** 10.3390/ijms140510539

**Published:** 2013-05-21

**Authors:** Qi Wang, Yongsheng Wang, Yue Li, Xuesong Gao, Shunai Liu, Jun Cheng

**Affiliations:** 1 Institute of Infectious Diseases, Beijing Ditan Hospital, Capital Medical University, Beijing 100015, China; E-Mails: wangqidl04@126.com (Q.W.); doctorwys@126.com (Y.W.); gaohappy@126.com (X.G.); liusa1031@sina.com (S.L.); 2 Beijing Key Laboratory of Emerging Infectious Diseases, Beijing 100015, China; 3 Institute of Materia Medica, Chinese Academy of Medical Sciences & Perking Union Medical College, Beijing 100050, China; 4 Beijing Center for Physical and Chemical Analysis, Beijing 100094, China; E-Mail: liyuedl04@126.com

**Keywords:** HCV, NS5A, NS5ATP9, cell proliferation, MEK/ERK

## Abstract

Hepatitis C virus (HCV) nonstructural protein 5A (NS5A) is a remarkable protein as it clearly plays multiple roles in mediating viral replication, host-cell interactions and viral pathogenesis. However, on the impact of cell growth, there have been different study results. NS5ATP9, also known as KIAA0101, p15PAF, L5, and OEACT-1, was first identified as a proliferating cell nuclear antigen-binding protein. Earlier studies have shown that NS5ATP9 might play an important role in HCV infection. The aim of this study is to investigate the function of NS5ATP9 on hepatocellular carcinoma (HCC) cell lines proliferation under HCV NS5A expression. The results showed that overexpression of NS5ATP9 inhibited the proliferation of Bel7402 cells, whereas knockdown of NS5ATP9 by interfering RNA promoted the growth of HepG2 cells. Under HCV NS5A expression, RNA interference (RNAi) targeting of NS5ATP9 could reverse the inhibition of HepG2 cell proliferation, suggesting that NS5ATP9 might be an anti-proliferation gene that plays an important role in the suppression of cell growth mediated by HCV NS5A via MEK/ERK signaling pathway. These findings might provide new insights into HCV NS5A and NS5ATP9.

## 1. Introduction

Hepatitis C virus (HCV) is one of the most common pathogens of chronic hepatitis. Persistent HCV infection often leads to liver cirrhosis and is associated with the development of hepatocellular carcinoma (HCC) [1,2]. However, research on the pathogenesis and viral replication as well as development of therapeutic strategies for control of HCV infections has been limited.

The HCV genome encodes a single polyprotein precursor that is cleaved by both host and viral proteases to generate at least four structural proteins (Core, E1, E2, and p7) and six nonstructural proteins (NS2, NS3, NS4A, NS4B, NS5A, and NS5B) [3]. HCV NS5A is a remarkable protein as it clearly plays multiple roles in mediating viral replication, host-cell interactions and viral pathogenesis [4]. HCV NS5A exists as multiple phospho-isoforms and is predominantly localized in the cytoplasmic/ perinuclear compartments of the cell, including the ER and the Golgi apparatus [4], and exists as two phosphoproteins (p56 and p58) with predominant phosphorylation on serine residues and a low level of phosphorylation on threonine residues [5]. HCV NS5A has been shown to interact with a wide variety of host cell proteins and thus may modulate lots of diverse signal transduction pathways relating to cell proliferation and cell-cycle control, apoptosis and cell survival, and cellular stress responses [6]. However, on the impact of cell growth, there have been different study results [7–13]. The further studies demonstrated that the expression of HCV NS5A could inhibit cell proliferation [12,13]. Overall, the mechanistic details of how HCV NS5A affects cell cycle control pathways are still not well understood and await further characterization in HCV infection system.

NS5ATP9 is also known as KIAA0101, p15PAF, L5, and OEACT-1. According to the NCBI database, the NS5ATP9 gene is located at 15q22.31 and its CDS consists of 336 bp that encode a 111-residue protein. The gene product of NS5ATP9 was first identified as a proliferating cell nuclear antigen (PCNA)-binding protein by yeast two-hybrid assay [14]. Recent studies have shown that NS5ATP9 expression is significantly elevated in some types of tumor tissues, but is down-regulated in others including HCC [14–21]. There were studies that suggested NS5ATP9 is involved in the regulation of diverse processes such as DNA repair, apoptosis, cellular signaling pathway, cell cycle and cell growth [14,16,17,22–26]. Our previous studies had shown that NS5ATP9 was up-regulated by HCV NS5A and that NF-κB bound to the NS5ATP9 promoter [27,28]. The recent study has also shown that KIAA0101 transcript variant 1 was overexpressed in HCC and could prevent doxorubicin-induced apoptosis by inhibiting p53 activation [20]. However, the effect was not definitely related to HCV.

In this study, we showed that NS5ATP9 overexpression inhibited Bel7402 cell proliferation, whereas knockdown of NS5ATP9 by RNAi promoted the growth of HepG2 cells. Under conditions of HCV NS5A expression, interfering RNA targeting of NS5ATP9 could reverse the inhibition of HepG2 cell proliferation, suggesting that NS5ATP9 could be an anti-proliferation gene that plays an important role in the suppression of cell growth mediated by HCV NS5A via MEK/ERK signaling pathway. This finding might provide new insights into the roles of HCV NS5A and NS5ATP9.

## 2. Results and Discussion

### 2.1. HCV NS5A Inhibited Proliferation of HCC Cell Lines

**Figure 1:**
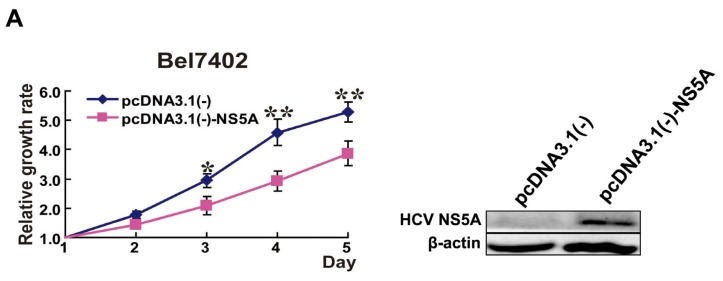

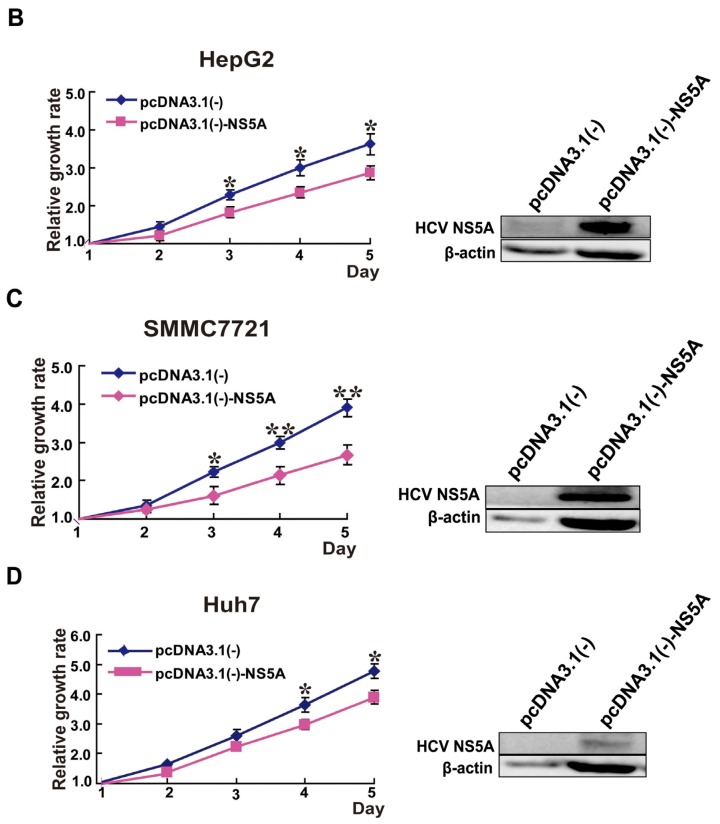
HCV NS5A inhibited proliferation of HCC cell lines. (Left) Cells were transfected with pcDNA3.1(−)-NS5A or pcDNA3.1(−). The relative growth rates were detected using cell viability assay. (Right) Cells were transfected with pcDNA3.1(−)- NS5A or pcDNA3.1(−). The protein levels of HCV NS5A were detected using Western blot after 24 h. (**A**) Bel7402 cell line; (**B**) HepG2 cell line; (**C**) SMMC7721 cell line; (**D**) Huh7 cell line. *p < 0.05, **p < 0.01; pcDNA3.1(−) *versus* pcDNA3.1(−)- NS5A.

In order to study the impact on cell growth by HCV NS5A, we used cell viability assay and found that HCV NS5A significantly inhibited proliferation of Bel7402, Huh7, SMMC7721, and HepG2 cell lines (Figure 1). The pictures of cell viability after transfection and during the experimental procedure were shown in Figure S1. Previous studies have shown that HCV NS5A promotes cell proliferation through a PKR-dependent mechanism [1,7] or down-regulation of p21WAF1 [8,11]. However, some studies showed that HCV NS5A expression actually inhibited cell proliferation in various cell types [12,13]. The underlying mechanism was suggested to be either p53-dependent induction of p21 [13], or through a p53-independent mechanism [12]. Arima *et al.* [13] reported that HCV NS5A-expressing human Chang liver, HeLa, and NIH3T3 cells all exhibit growth retardation compared with the control cells. However, the molecular signaling pathway involved remains largely unknown. Researchers haven’t explained the underlying reasons for the opposite conclusions, but might be due to the different methods or techniques used by researchers, or the different types of tissues or cells. In this study, we found that HCV NS5A significantly inhibited proliferation of HCC cell lines. These results are consistent with the previous reports of inhibition effect.

### 2.2. Four HCC Cell Lines Showed the Differential mRNA Levels of NS5ATP9

**Figure 2:**
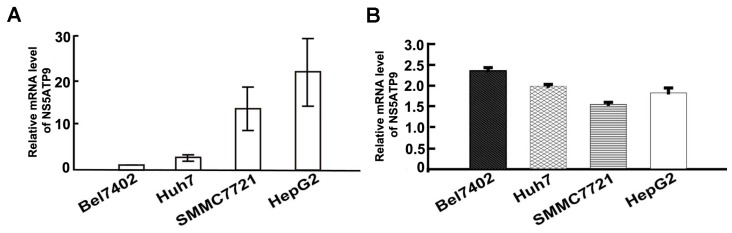
Relative mRNA levels of NS5ATP9 were detected using Real time PCR. (**A**) Real time PCR validated that NS5ATP9 was differently expressed in four HCC cell lines. Data were analyzed using the 2^−ΔΔ^*^C^*^t^ method. Briefly, the differential expression of the NS5ATP9 gene *versus* the housekeeping gene was first calculated using the expression: Δ*C*t = *C*t _NS5ATP9_ – *C*t _G3PDH_. Next, other cell lines: Bel7402 cell line ratios were calculated from the Δ*C*t values = 2^−(Δ^*^C^*^t cell line − Δ^*^C^*^t Bel7402)^; (**B**) Real time PCR was used to detect relative mRNA levels of NS5ATP9 in response to HCV NS5A expression. pcDNA3.1(−)-NS5A was transfected into Bel7402, Huh7, SMMC7721, and HepG2 cells. Real time PCR data were also analyzed using the 2^−ΔΔ^*^C^*^t^ method. Briefly, the differential expression of the NS5ATP9 *versus* G3PDH was first calculated using the expression: Δ*C*t = *C*t _NS5ATP9_ – *C*t _G3PDH_. Next, pcDNA3.1(−)-NS5A: pcDNA3.1(−) ratios were calculated from the Δ*C*t values = 2^−[Δ^*^C^*^t pcDNA3.1(−)-NS5A – Δ^*^C^*^t pcDNA3.1(−)]^.

To determine the expression patterns of NS5ATP9 in HCC cell lines, we used Real time PCR for measurement of the mRNA expression of NS5ATP9. We detected NS5ATP9 mRNA in all four cell lines, although the expression pattern varied. Among the cell lines examined, the Bel7402 cell line had the lowest level of NS5ATP9 expression; the expression being 2.46-, 12.04- and 19.29-fold lower when compared with the Huh7, SMMC7721, and HepG2 cell lines, respectively (Figure 2A). The fold induction values were calculated using the 2^−ΔΔ^*^C^*^t^ method.

### 2.3. HCV NS5A Up-Regulated NS5ATP9 mRNA Levels in HCC Cell Lines

Our previous study showed that NS5ATP9 is up-regulated by HCV NS5A via NF-κB binding to the NS5ATP9 gene promoter. Early studies showed that NS5ATP9 expression was significantly changed in some types of tumors [14–18]. However, whether and how NS5ATP9 participates in the regulation of the biological effects mediated by HCV NS5A is not clearly understood. Therefore, there is a need to further investigate the role of NS5ATP9 in cell behavior.

In this study, Real time PCR was used to detect the mRNA levels of NS5ATP9 in response to HCV NS5A. Plasmid DNA of pcDNA3.1(−)-NS5A was transfected into Bel7402, Huh7, SMMC7721, and HepG2 cells. Cells transfected with pcDNA3.1(−)were used as controls. Real time PCR data were expressed as T:N ratios [T: transfected with pcDNA3.1(−)-NS5A, N: transfected with pcDNA3.1(−)], calculated using the 2^−ΔΔ^*^C^*^t^ method after normalization to G3PDH. The NS5ATP9 mRNA levels were induced by 2.3-, 1.9-, 1.6-, and 1.8-fold in Bel7402, Huh7, SMMC7721, and HepG2 cells, respectively (Figure 2B).

### 2.4. Overexpression and Knockdown of NS5ATP9 Showed the Reverse Effect on Cell Proliferation

**Figure 3:**
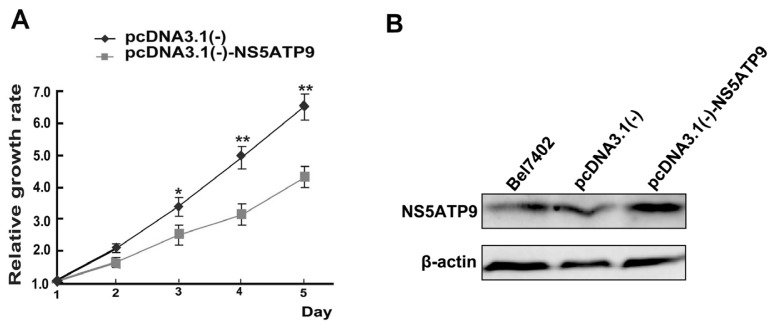
NS5ATP9 overexpression inhibited Bel7402 cell proliferation. (**A**) The relative growth rates of Bel7402 cells transfected with pcDNA3.1(−)-NS5ATP9 or pcDNA3.1(−) were detected using cell viability assay; (**B**) The protein levels of NS5ATP9 were observed by Western blot when pcDNA3.1(−)-NS5ATP9 and pcDNA3.1(−) were transfected into Bel7402 cells, respectively. *p < 0.05, **p < 0.01; pcDNA3.1(−) *versus* pcDNA3.1(−)- NS5ATP9.

**Figure 4:**
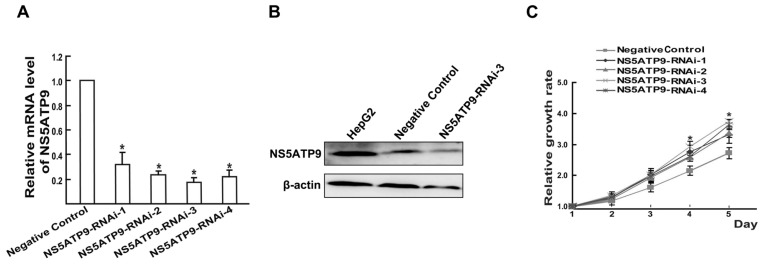
NS5ATP9 knockdown promoted HepG2 cell proliferation. (**A**) The relative mRNA levels of NS5ATP9 were observed by Real time PCR when NS5ATP9-RNAi was transfected into HepG2 cells, respectively; (**B**) The protein levels of NS5ATP9 were observed by Western blot when Negative Control and NS5ATP9-RNAi-3 were transfected into HepG2 cells, respectively; (**C**) The relative growth rates of HepG2 cells transfected with NS5ATP9-RNAi or Negative Control were detected using cell viability assay. The results showed that NS5ATP9 knockdown promoted HepG2 cell proliferation. *p < 0.05; Negative Control *versus* NS5ATP9-RNAi.

In order to investigate the biological significance of NS5ATP9, we selected the Bel7402 cell line (which had the lowest mRNA level of NS5ATP9) for NS5ATP9 overexpression, and the HepG2 cell line (which had the highest mRNA level of NS5ATP9) for knockdown. Significant effects of overexpression and knockdown were observed when pcDNA3.1(−)-NS5ATP9 and NS5ATP9-RNAi-3 were transfected into Bel7402 and HepG2 cells, respectively. The NS5ATP9 protein level in Bel7402 cells transfected with pcDNA3.1(−)-NS5ATP9 was approximately higher than that of cells transfected with pcDNA3.1(−) (Figure 3B). The mRNA levels of NS5ATP9 in HepG2 cells were reduced to 32%, 23%, 18% and 22%, respectively, by the different NS5ATP9-RNAi vectors (Figure 4A). And the NS5ATP9 protein level in cells transfected with NS5ATP9-RNAi-3 was lower than in cells transfected with Negative Control (Figure 4B).

Cell viability assay was used to examine the proliferative capacity of Bel7402 cells (Figure 3A) and HepG2 cells (Figure 4C). The results showed that NS5ATP9 overexpression significantly inhibited Bel7402 cell proliferation, whereas knockdown of NS5ATP9 promoted HepG2 cell proliferation, indicating that NS5ATP9 is likely to play an anti-proliferation role in these cells.

### 2.5. NS5ATP9 Mediated the Inhibition of Proliferation under HCV NS5A Expression

**Figure 5:**
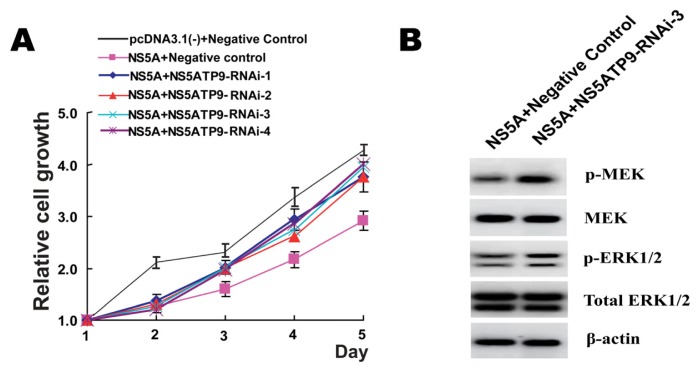
MEK/ERK signaling pathway is involved in NS5ATP9-mediated proliferation inhibition under HCV NS5A expression. (**A**) Co-transfection with pcDNA3.1(−)-NS5A and NS5ATP9-RNAi promoted cell proliferation compared with co-transfection of pcDNA3.1(−)-NS5A and Negative Control or pcDNA3.1(−) and Negative Control; (**B**) Western blot was used to detect the expression and phosphorylation levels of MEK and ERK. Beta-actin was selected as a control. The results showed that the protein levels of MEK and total ERK in cells co-tansfected with pcDNA3.1(−) -NS5A and NS5ATP9- RNAi-3 were not significantly different from the cells co-tansfected with pcDNA3.1(−) -NS5A and Negative Control; however, the levels of p-MEK and p-ERK1/2 were significantly elevated.

In order to investigate the role of NS5ATP9 in the inhibition of cell growth by HCV NS5A, we examined HepG2 cell proliferation under conditions of RNAi-mediated down-regulation of NS5ATP9 and HCV NS5A expression. The high-efficiency transfection was obtained in this study, represented as the ratio of GFP-positive cells in Figure S2. The data confirmed that co-transfection with pcDNA3.1(−)-NS5A and NS5ATP9-RNAi promoted HepG2 cell proliferation when compared with co-transfection of pcDNA3.1(−)-NS5A and Negative Control or pcDNA3.1(−) and Negative Control (Figure 5A).

Dependent on the results, we found that NS5ATP9 knockdown could reverse the inhibition of HepG2 cell proliferation caused by HCV NS5A expression, clearly indicating the role of NS5ATP9 in the suppression of cell growth mediated by HCV NS5A. These results support the contention that NS5ATP9 plays a role in HCC cell proliferation, which is consistent with the results from previous research [18].

### 2.6. NS5ATP9 Knockdown Activated MEK/ERK Signaling Pathway under HCV NS5A Expression

Up-regulation of cellular suppressor genes may be a mechanism for disrupting cell growth [13]. Many studies have been devoted to elucidating the biochemical activities of the extracellular signal-regulated kinase (ERK) pathways and their relationships within the cascade. Mitogen-activated protein kinase (MAPK) cascades are crucial signaling pathways involved in the regulation of normal cell proliferation, survival, and differentiation. ERK is a key downstream component of MAPK cascades and Mitogen-activated protein kinase kinase (MEK) is the specific kinase that phosphorylates ERK [13]. Research in recent years has revealed that, in addition to the plasma membrane, ERK signaling could occur in other intracellular compartments, and that numerous auxiliary factors, such as ERK scaffolding proteins and signaling modulators, play key roles in determining the strength, duration, and location of ERK signaling [29]. Georgopoulou *et al.* [30] demonstrated that the HCV NS5A protein interacts with the growth receptor-bound protein 2 (Grb2) and inhibits the phosphorylation of the extracellular signal-regulated kinases 1 and 2 (ERK1/2) in HeLa, NIH3T3 or liver cells.

In this study, Western blot was used to detect the expression levels of MEK and ERK in HepG2 cells co-tansfected with pcDNA3.1(−)-NS5A and NS5ATP9-RNAi-3, which produced the highest rate of interference, and cells co-tansfected with pcDNA3.1(−)-NS5A and Negative Control were used as a control. We observed that at 72 h after co-transfection, the protein levels of MEK and total ERK were not significantly different from the control; however, the phosphorylation status of MEK and ERK (p-MEK and p-ERK1/2) were significantly elevated (Figure 5B), suggesting that the MEK/ERK signaling pathway was activated.

Our results demonstrate that NS5ATP9 accounted for the suppression of cell growth by HCV NS5A through interfering with MEK/ERK signaling pathway, at least partially. Since NS5ATP9 can interact with certain cellular molecules related to cell proliferation, such as PCNA and p33ING1b [14,23], we speculated that NS5ATP9 may interact with MEK and/or ERK molecules directly or with molecules that regulate the MEK/ERK signaling pathway, thereby resulting in MEK/ERK signaling pathway chaos and disturbance of cell behavior. However, details of the underlying molecular mechanism still need to be further analyzed. Elucidating the details of these events and whether NS5ATP9 can directly interact with MEK and/or ERK or other molecules of this cascade will be an important step in understanding how HCV NS5A and NS5ATP9 mediate cell proliferation inhibition, and may provide valuable information for therapeutic intervention against HCV infection and HCC.

## 3. Experimental Section

### 3.1. Cell Culture and Transient Transfection

A human hepatoma cell line, Huh-7, was established from a hepatocellular carcinoma in 1982 [31]. SMMC7721 and Bel 7402 are all derived from different specimens for primary liver cell carcinomas [32]. HepG2 is a kind of human hepatoma-derived cell line [33]. In this study, Bel7402 and SMMC7721 cell lines were purchased from Chinese Academy of Science Cell Bank (www.cellbank.org.cn), and HepG2 and Huh7 cell lines were preserved in our laboratory. They were all cultured in Dulbecco’s modified Eagle’s medium (DMEM; Gibco, Carlsbad, CA, USA) containing 10% fetal bovine serum (FBS; Gibco, Carlsbad, CA, USA), 100 U/mL of penicillin, and 100 μg/mL of streptomycin. All of them were cultured in a humidified chamber at 37 °C in 5% CO_2_. Cells were transient transfected using Lipofectamine 2000 (Invitrogen, Carlsbad, CA, USA) according to the manufacturer’s instructions.

### 3.2. Expression and RNAi Plasmids Construction

Full-length sequences of HCV NS5A (1b genotype) and NS5ATP9 were amplified and subcloned into pcDNA3.1(−) using EcoRI and BamHI, respectively[27]. A pcDNA™6.2-GW/EmGFP-miR vector (Invitrogen) was used for DNA vector-based siRNA synthesis under the control of an RNA polymerase II-dependent promoter. The vector was constructed using Invitrogen’s online RNAi Designer (www.invitrogen.com/rnai) [34], targeting NS5ATP9 (GenBank accession number: NM_014736). Four pairs of complementary single-stranded DNA oligonucleotide were synthesized, and the four forward sequences as follows:

NS5ATP9-RNAi-1(5′-TGCTGAACACTGTCTGCTTTAGTCCGGTTTTGGCCACTGACTGACCG GACTAACAGACAGTGTT-3′);
NS5ATP9-RNAi-2(5′-TGCTGTTCTGTAAGTGCCTGGAACACGTTTTGGCCACTGACTGACGT GTTCCACACTTACAGAA-3′);
NS5ATP9-RNAi-3(5′-TGCTGACTGATGTCGAATTAGTGGCAGTTTTGGCCACTGACTGACTG CCACTATCGACATCAGT-3′);
NS5ATP9-RNAi-4(5′-TGCTGTTTCCTAAGCCACTGCTTCCTGTTTTGGCCACTGACTGACAGG AAGC AGG CTTAG GAAA-3′).

The paired single-stranded oligonucleotides were annealed to generate a double-stranded oligo. Then the double-stranded oligos were cloned into the linearized pcDNA™6.2-GW/EmGFP-miR vectors to construct NS5ATP9-RNAi plasmids which were used for NS5ATP9 knockdown in cells. The Negative Control plasmid (5′-TGCTGAAATGTACTGCGCGTGGAGACGTTTTGGCCACTGACTGACGTCT CCACGCAGTACATTT-3′), which was predicted not to target any known vertebrate gene, was purchased from Invitrogen for detecting nonspecific effect. Cells were transfected using Lipofectamine 2000 (Invitrogen) according to the manufacturer’s instructions.

### 3.3. Real Time PCR Analysis

Total RNA was extracted using TRIzol reagent (Invitrogen). A SYBR ExScript RT-PCR Kit (TaKaRa, Dalian, China) was used for Real time PCR. Reactions for each sample were performed in triplicate with equal amounts of template cDNA using the Roche Lightcycler system and Lightcycler 3 software. The sense and anti-sense primers for NS5ATP9 were 5′-ATGGTGCGGACTA AAGCAGAC AG-3′ and 5′-TGTCGAATTAGTGGCAGAGGTGG-3′, respectively. G3PDH was amplified simultaneously as an internal control using sense (5′-CCTGTTCGACAGTCAGCCG-3′) and anti-sense (5′-CGACCAAATCCGTTGACTCC-3′) primers. The Real time PCR conditions for amplifying NS5ATP9 and G3PDH were as follows: 95 °C for 10 s, followed by 40 cycles at 95 °C for 5 s and 60 °C for 20 s. Fold induction values were calculated using the 2^ΔΔ^*^C^*^t^ method according to the manufacturer’s instructions.

### 3.4. Cell Viability Assay

Cell viability assay experiments were performed using a 96-well format in triplicate using the Promega CellTiter Glo^®^ ATP-based assay, according to the manufacturer’s instructions. In brief, the plate was equilibrated to room temperature for approximately 30 min at indicated time points. An equal volume of CellTiter-Glo^®^ Reagent was added to the cell culture present in each well. The contents were mixed for 2 min on an orbital shaker to induce cell lysis. The plate was then incubated at room temperature for 10 min to stabilize the luminescent signal and, finally, the luminescence was recorded using a Veritas Microplate Luminometer (Turner BioSystems, Sunnyvale, CA, USA).

### 3.5. Western Blot

Cells cultured in 6-well plate were lysed in a radioimmunoprecipitation assay buffer [50 mM HEPES (pH 7.4), 150 mM NaCl, 1% Triton-X-100, 1% deoxycholate, 0.1% SDS] containing proteinase inhibitors (Sigma, St. Louis, MO, USA). The debris was discarded by centrifuging for 20 min at 14,000× *g* and the supernatants containing total protein were quantified with a standard protein assay (Pierce, Rockford, IL, USA). Samples were analyzed by SDS-PAGE and transferred to PVDF membranes (Millipore, Bedford, MA, USA). After blocked with 5% skim milk, the membranes were probed with primary antibodies of ERK1/2, total-ERK1/2, MEK, p-MEK, HCV NS5A (Abcam, Cambridge, UK), β-actin, or NS5ATP9 (Santa Cruz, Santa Cruz, CA, USA) at optimum dilutions followed by appropriate secondary antibodies, and the immunoreactive signals were detected using an Enhanced Chemiluminescence kit (Pierce, Rockford, IL, USA) through an ECL system.

### 3.6. Statistical Analysis

All experiments were repeated at least three times. Data were expressed as means ± SD. Differences between experimental groups were assessed using the two-tailed *t*-test. *p* < 0.05 was considered statistically significant.

## 4. Conclusions

We demonstrated that overexpression of NS5ATP9 inhibited the proliferation of Bel7402 cells, whereas knockdown of NS5ATP9 by interfering RNA promoted the growth of HepG2 cells. Under conditions of HCV NS5A expression, RNAi targeting of NS5ATP9 could reverse the inhibition of HepG2 cell proliferation, suggesting that NS5ATP9 might be an anti-proliferation gene that plays an important role in the suppression of cell growth mediated by HCV NS5A via MEK/ERK signaling pathway.

## Abbreviations

HCV NS5A: Hepatitis C virus nonstructural protein 5A
HCC: hepatocellular carcinoma
RNAi: RNA interference
β-actin: beta-actin
MAPK: mitogen-activated protein kinase
MEK: mitogen-activated protein kinase kinase
ERK: extracellular signal regulated kinase.
